# Junctional Zone in Infertile Women: A Three-dimensional Ultrasound Study

**DOI:** 10.1055/s-0040-1708089

**Published:** 2020-03

**Authors:** Vanessa Silva, Flávia Fundora Ramos, Ana Filipa Matos Brás, Ricardo Filipe Sousa Santos, Maria Sofia Dantas Pinto Lobo Xavier, Rui Filipe Oliveira Miguelote

**Affiliations:** 1 Service of Gynecology and Obstetrics, Centro de Procriação Medicamente Assistida, Hospital Senhora da Oliveira, Guimarães, Portugal; 2 Escola de Medicina, Instituto de Investigação em Ciências da Vida e Saúde, Universidade do Minho, Braga, Portugal; 3 Escola de Medicina, Universidade do Minho, Braga, Portugal; 4 Department of Community Medicine and Health Decision, Faculdade de Medicina, Centro de Investigação em Tecnologias e Serviços de Saúde, Universidade do Porto, Porto, Portugal

**Keywords:** 3D ultrasound, junctional zone, reproducibility, infertility, associated factors, ecografia 3D, zona juncional, reprodutibilidade, infertilidade, factores associados

## Abstract

**Objective** To analyze the interobserver and intraobserver reproducibility of the visualization and continuity of the juncional zone (JZ) by three-dimensional (3D) ultrasound in infertile women, and to evaluate the sociodemographic, hormonal, and structural factors that influence these assessments.

**Methods** A prospective study conducted at the Assisted Reproductive Technology Unit of Hospital Senhora da Oliveira, in the city of Guimarães, Portugal. Transvaginal 3D ultrasonography was performed, and 2 volumes were generated per case. Two observers who were blinded to each other's work analyzed these volumes, choosing the best coronal section. Four months later, one of the observers performed the same methodology. The JZ visualization was classified as optimal, satisfactory, and unsatisfactory, and the JZ continuity, as continuous and discontinuous. The interobserver and intraobserver agreements were analyzed. The influence of hormonal, structural, and sociodemographic factors on the JZ was evaluated.

**Results** In total, 65 women were included in the present study. The interobserver reproducibility was substantial for JZ visualization and continuity (k = 0.635 and 0.753 respectively), and the intraobserver reproducibility was very good for JZ visualization and continuity (k = 0.884 and 0.816 respectively). Trilaminar endometrial pattern was associated with optimal JZ visualization (*p* = 0.012). The increase of 1 unit in the level of serum estradiol represents a 9.9% decrease in the odds of unsatisfactory visualization of the JZ (odds ratio [OR] = 0.9; 95% confidence interval [95%CI] = 0.814–0.996; *p* = 0.042). Endometriosis increases the odds of unsatisfactory visualization by 24 times (OR = 23.7; 95%CI = 1.262–437.057; *p* = 0.034). The prevalence of discontinuous JZs was of 60%. Myomas and endometriosis were associated with discontinuous JZs (*p* = 0.034 and 0.016 respectively).

**Conclusion** The assessment of JZ visualization and continuity by 3D ultrasound is reproducible enough to be used in the clinical practice.

## Resumo

**Objetivo** Analisar a reprodutibilidade inter e intraobservador da visualização e continuidade da zona juncional (ZJ) por ecografia tridimensional (3D) em mulheres inférteis, e avaliar os fatores sociodemográficos, hormonais e estruturais que afetam essas avaliações.

**Métodos** Um estudo prospectivo conduzido no Centro de Procriação Medicamente Assistida do Hospital Senhora da Oliveira, em Guimarães, Portugal. Foi realizada ecografia transvaginal 3D e gerados 2 volumes por caso. Dois observadores, cegos às avaliações um do outro, analisaram os volumes obtidos e escolheram o melhor corte coronal. Após quatro meses, a mesma análise foi realizada por um dos observadores. A visualização da ZJ foi classificada como ótima, satisfatória e não satisfatória, e a continuidade, como contínua ou descontínua. Foram avaliadas as reprodutibilidades inter e intraobservador. A influência de fatores sociodemográficos, hormonais e estruturais na ZJ foi analisada.

**Resultados** No total, 65 mulheres foram incluídas no presente estudo. A reprodutibilidade interobservador foi substancial para a visualização e continuidade da ZJ (k = 0,635 e 0,753, respetivamente). A reprodutibilidade intraobservador foi muito boa para a visualização e continuidade da ZJ (k = 0,884 e 0,816, respetivamente). Endométrio trilaminar associou-se à visualização ótima da ZJ (*p* = 0.012). O aumento de 1 unidade no nível de estradiol diminuiu a chance de visualização não satisfatória da ZJ em 9,9% (razão de probabilidades [RP] = 0,9; intervalo de confiança de 95% [CI95%] = 0,814–0,996; *p* = 0,042). Endometriose aumentou a chance de visualização não satisfatória da ZJ em 24 vezes (RP = 23,7; CI95% =  1,262–437,057; *p* = 0,034). A prevalência de ZJs descontínuas foi de 60%. Miomas e endometriose associaram-se a ZJs descontínuas (*p* = 0,034 e 0,016, respetivamente).

**Conclusão** A avaliação da visualização e continuidade da ZJ por ecografia 3D é reprodutível, podendo ser utilizada na prática clínica.

## Introduction

The junctional zone (JZ) is a hormone-dependent zone that corresponds to the inner layer of the myometrium, which derives from the paramesonephric ducts. In contrast, the outer myometrium has a different embryonic origin – a mesenchymal one.^1^ The JZ contains myocytes as the outer myometrium, but with different morphologic characteristics. Junctional-zone myocytes have a higher nuclear-cytoplasmic ratio, reduced extracellular matrix, low content of water, and a concentric architectural arrangement.^1^ These characteristics enable the identification of the JZ on ultrasound.^1^

The JZ plays an important role in reproduction and pregnancy. Uterine contractility in non-pregnant women originated exclusively in the JZ changes in orientation, amplitude, and frequency according to the phase of the menstrual cycle.^2^ ^3^ These contractions increase and take the direction of the cervix to the uterine fundus in the follicular and periovulatory phase, facilitating sperm transport.^2^ ^4^ ^5^ Peristaltic activity decreases in the luteal phase, facilitating the implantation, oxygenation, and nutrition of the blastocyst.^2^ ^6^

When uterine contractility is excessive and uncoordinated, it is associated with lower pregnancy rates both in natural and assisted reproductive technology cycles.^7^ ^8^ Studies^2^ ^9^ ^10^ show that a dysfunctional JZ plays an important role in the physiopathology of endometriosis, facilitating retrograde menstruation and implantation of endometrial cells in the pelvis. On the other hand, JZ characteristics observed by three-dimensional (3D) ultrasound and magnetic resonance imaging (MRI), such as increased thickness and disruption, are associated with endometriosis, but also with adenomyosis.^11^ ^12^

Several studies^1^ ^13^ ^14^ suggest that the assessment of the JZ by 3D transvaginal ultrasound should be included in routine uterine ultrasound. Through the coronal plane, it is possible to delimit the JZ at the fundus and lateral walls, which is observed as a thin and regular layer with hypoechogenic characteristics between the basal layer of the endometrium and the outer myometrium.^1^ ^15^ Despite growing research involving the evaluation of the JZ by 3D ultrasound, there is no universally accepted methodology.

Due to the cost-benefit ratio, convenience and availability of 3D ultrasound, and the importance of the JZ, particularly in infertile women, we aim to evaluate the interobserver and intraobserver reproducibility of the visualization and continuity of the JZ by 3D ultrasound as well as to identify the sociodemographic and physiological factors that affect the JZ.

## Methods

A prospective observational study was conducted with women followed at the Assisted Reproductive Technology Unit of Hospital Senhora da Oliveira, in the city of Guimarães, Portugal, from July to October of 2018. The exclusion criteria were serum follicle-stimulating hormone levels greater than 25UI/L, women older than 40 years of age, and cases with incomplete data on the ultrasound evaluation (absence of 2 volumes per case). One of the authors (VS) acquired the planes and volumes through transvaginal ultrasound (with the woman in the lithotomy position and an empty bladder), using a Samsung (Seoul, South Korea) HS50 ultrasound with a 5–9 MHz 2D/3D endocavitary probe. All evaluations were performed using a standardized methodology, as explained below.

**Fig. 1:**
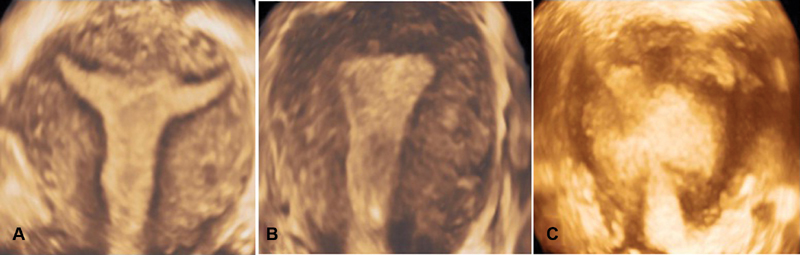
Three-dimensional ultrasound imaging of optimal (**A**), satisfactory (**B**) and unsatisfactory (**C**) visualizations of the JZ in the coronal plane.

For the uterine assessment, two-dimensional (2D) transverse and longitudinal planes were performed. The uterus was centered on the screen. In the longitudinal plane, the endometrial thickness was measured (in the double layer, in its thickest zone) parallel to the anteroposterior diameter of the uterus. In the presence of intracavitary fluid, the endometrial thickness was obtained by subtracting the thickness of the intracavitary fluid from the total thickness of the endometrial cavity.^16^ The anomalies observed, such as myomas or adenomyosis, were identified according to established diagnostic criteria.^17^ The pelvic cavity was explored for signs of endometriosis (endometriomas or deep endometriosis with the involvement of the bladder, bowel, or rectovaginal septum).^18^ After the 2D ultrasound, a 3D ultrasound was performed. In the longitudinal plane, the uterus was centered on the screen so that the cervical canal and the myometrium were fully visible. The 3D acquisition box was adjusted to include a margin around the uterus, which allowed delimitation of the myometrium. A maximal image quality and an acquisition angle (90°) were selected. The probe was held firmly, and the women were asked to hold their breath during acquisition to minimize the artifacts. After the acquisition, a summary evaluation of the volume was performed, and whenever it turned out to be of poor quality (a volume that did not enable the complete assessment of the uterine volume or the presence of movement artifacts), new ones were obtained. Two volumes per case were obtained from each participant, and the volumes were stored in the ultrasound memory. An analysis of the 3D volumes was performed posteriorly, using the Samsung Medison 5D Viewer software. The methodology of 3D image postprocessing and classification of JZ visualization and continuity was initially standardized between the authors (VS and FR) in a set of volumes not included in the study sample. The multiplanar image was freely adjusted for the JZ assessment to obtain a coronal plane with adequate visualization of the endometrial cavity, the external contour of the uterus, and the interstitial portions of the fallopian tube. A region of interest (ROI) box with any rendering mode combination was used to optimize the visualization of the JZ. The best reconstructed coronal image was chosen for each participant from the two volumes acquired per operator. The visualization of the JZ was classified as proposed by Naftalin et al^14^ (Fig. 1):

- Optimal: JZ clearly visible and evaluable in its entirety.
- Satisfactory: most, but not all of the JZ can be clearly observed
- Unsatisfactory: a great portion of the JZ could not be clearly observed.^14^

**Fig. 2:**
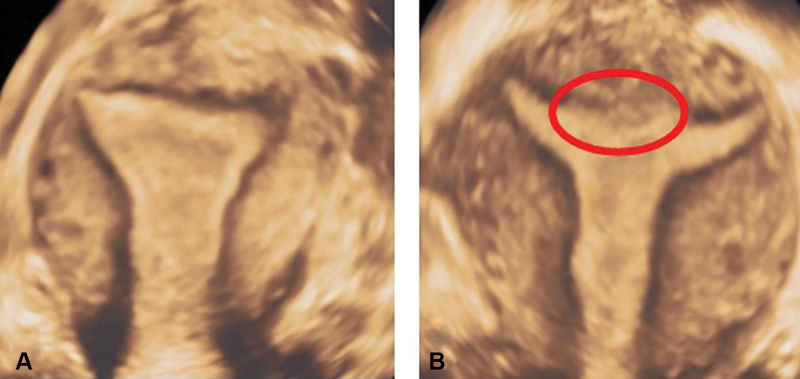
Three-dimensional ultrasound imaging of continuous (**A**) and discontinuous (**B**) JZ in the coronal plane. Note the discontinuous JZ on the coronal section, marked in (**B**) by the red circle.

The continuity of the JZ was classified as proposed by Van den Bosch et al^17^ (Fig. 2):

- Continuous: uninterrupted JZ.
- Discontinuous: JZ with signs of disruption.^17^

Two authors (VS and FR) performed the postprocessing and analysis of the 3D volumes independently and blindly. Both authors classified the best coronal plane of the two volumes acquired per participant to determine the interobserver agreement. Four months after the first analysis, VS reassessed the volumes (two volumes per case, choosing the best reconstructed coronal image from the two volumes acquired) to determine the intraobserver agreement. The intraobserver and interobserver agreements were assessed for visualization and continuity of the JZ.

Demographic and physiological (hormonal and structural) variables, as well as gynecological antecedents were collected by consulting the participants' electronic clinical process and 2D/3D transvaginal ultrasound. Then their effects on the visualization and continuity of the JZ were evaluated.

Data analysis was performed using the Statistical Package for Social Sciences (SPSS, IBM Corp., Armonk, NY, US), version 25.0. The interobserver and intraobserver agreements were assessed using the Cohen k, which was interpreted as poor (k < 0.20), reasonable (k between 0.21 and 0.40), moderate (k between 0.41 and 0.60), good (k between 0.61 and 0.80), and very good (k between 0.81 and 1.00).^19^ Rejection of the null hypothesis was based on the significance level of *p* < 0.05. The cases of unsatisfactory visualization were excluded from the assessment of continuity of the JZ, and most cases of disagreement in the classification of visualization of the JZ (unsatisfactory versus satisfactory/optimal) were solved. The unsolved cases of disagreement were excluded from the analysis.

A descriptive analysis was performed taking into account the symmetry of the distribution of the continuous variables. Based on the result of the Shapiro-Wilk test, parametric or nonparametric statistics were used according to *p* > 0.05 or *p* < 0.05. The Chi-squared (χ^2^) test or the Fisher exact test were used to evaluate the association between visualization or continuity of the JZ and the categorical variables. Analysis of variance (ANOVA) and *t*-tests were used to analyze the continuous variables with normal distribution. The continuous variables without normal distribution were compared using the Kruskall-Wallis and Mann-Whitney tests. Additionally, the effect on the visualization and continuity of the JZ was evaluated through univariate and multivariate analyses. Cases of intraobserver and interobserver disagreement were excluded from the analysis. Cases of unsatisfactory visualization were excluded to evaluate the factors that influence the continuity of the JZ.

The Health Ethics Committee of Hospital Senhora da Oliveira approved the research project. The participants signed an informed consent form to participate in the study.

## Results

### Sample

In total, 65 women between 23 and 40 years of age were included in the present study (mean [M] = 33.01; standard deviation [SD] = 4.27), and 24 (36.9%) had irregular cycles, 28 (43.1%) were in the follicular phase of the menstrual cycle, and 43 (66.2%) had a trilaminar endometrial pattern. No cases of adenomyosis were reported, and 15 (23.1%) of the participants had diagnosed or suspected endometriosis. The results of the remaining descriptive analysis are in Table 1.

**Table 1:** Descriptive analysis of the characteristics of the sample (*n* = 65)

Variables	
Age (years)	33.01(4.27)
Body mass index (kg/m^2^)	25.8(5.39)
Menarche (years)	12.66(1.66)
Parity	
Nullipara	51(78.5)
Regularity of cycle	
Irregular	24(36.9)
Regular	41(63.1)
Stage of the cycle	
Proliferative	28(43.1)
Secretory	13(20.0)
Previous uterine surgery	3(4.6)
Follicle-stimulating hormone (IU/L)	7.20(3.77)
Luteinizing hormone (IU/L)	4.77(2.46)
Estradiol (pg/mL)	61.15(33.58)
Total testosterone (ng/dL)	26.09(10.61)
Prolactin (µg/L)	16.79(29.12)
Endometrium thickness (mm)	7.18(3.06)
Endometrium thickness (≤5 mm)	16(24.6)
Endometrial ultrasound characteristics	
Trilaminar	43(66.2)
Heterogeneous	22(33.8)
Mioma	10(15.4)
Diagnosed or suspected endometriosis	16(24.6)
Adenomyosis	0(0)

Results presented as M (SD) and n(%).

Abbreviations: M, average; SD, standard deviation; n, absolute frequency; %, relative frequency; dl, deciliter; IU, international units; L, liter; pg, picogram; mL, mililiter; mm, milimeter.

### Interobserver and Intraobserver Agreements Regarding JZ Visualization

Regarding the visualization of the JZ, the interobserver agreement was good, with a Cohen k of 0.635. The total agreement was of 80.0%. The highest number of disagreements occurred between cases of satisfactory and optimal visualization (17.2%). The intraobserver agreement was very good, with a Cohen k of 0.884, and the total agreement was of 93.9%. Again, the highest number of disagreements occurred between cases of satisfactory and optimal visualization (4.6%). Details of the analysis are in Tables 2 and 3.

**Table 2:** Visualization of the junctional zone and interobserver agreement (*n* = 65; observers: FR and VS)

	FR		
VS	Unsatisfactory n(%)	Satisfactory n(%)	Optimal n(%)
**Unsatisfactory n(%)**	6(9.2)	1(1.5)	0(0)
**Satisfactory n(%)**	2(3.1)	12(18.5)	3(4.6)
**Optimal n(%)**	0(0)	7(12.6)	34(52.3)
**Absolute agreement**	Cohen k = 0.635		

**Table 3:** Visualization of the junctional zone and intraobserver agreement (*n* = 65)

	2^nd^ assessment		
1^st^ assessment	Unsatisfactory n(%)	Satisfactory n(%)	Optimal n(%)
**Unsatisfactory n(%)**	7(10.8)	0(0)	0(0)
**Satisfactory n(%)**	1(1.5)	15(23.1)	1(1.5)
**Optimal n(%)**	0(0)	2(3.1)	39(60.0)
**Absolute agreement**	Cohen k = 0.884		

### Interobserver and Intraobserver Agreements Regarding JZ Continuity

Regarding the continuity of the JZ, the interobserver agreement was good, with a Cohen k of 0.753. The total agreement was of 88.1%. The intraobserver agreement was very good, with a Cohen k of 0.816, and a total agreement of 91.2%. Details of the analysis are in Tables 4 and 5.

**Table 4:** Continuity of the junctional zone and interobserver agreement (*n* = 59*; observers: VS and FR)

	FR	
VS	Continuous n(%)	Discontinuous n(%)
**Continuous n(%)**	20(33.9)	2(3.4)
**Discontinuous n(%)**	5(8.5)	32(54.2)
**Absolute agreement**	Cohen k = 0.753	

Note: *Excluding cases of unsatisfactory assessment of the junctional zone (*n* = 6) and visualization disagreements (unsatisfactory versus satisfactory/optimal; *n* = 0).

**Table 5:** Continuity of the junctional zone and intraobserver agreement (*n* = 57*)

	2^nd^ assessment	
1^st^ assessment	Continuous n(%)	Discontinuous n(%)
**Continuous n(%)**	20(35.1)	2(3.5)
**Discontinuous n(%)**	3(5.3)	32(56.1)
**Absolute agreement**	Cohen k = 0.820	

Note: *Excluding cases of unsatisfactory assessment of the junctional zone(*n* = 6) and visualization disagreements (unsatisfactory versus satisfactory/optimal; *n* = 2).

### Factors Affecting JZ Visualization

We tried to identify variables that influenced the visualization of the JZ. In order to do so, cases of interobserver and intraobserver disagreement were solved. We excluded five cases without a final agreement. Regarding the visualization of the JZ among the 60 remaining cases, 65% (39/60) of the women had optimal, 25% (15/60) had satisfactory, and 10% (6/60) had unsatisfactory visualization. Serum estradiol levels, endometrial ultrasound appearance, and diagnosed or suspected endometriosis influenced the visualization of the JZ (Table 6). Higher estradiol levels were associated with better visualization of the JZ (satisfactory and optimal JZ, *p* = 0.016), particularly in the comparison between unsatisfactory and optimal JZ assessment (*p* = 0.013). On the other hand, the presence of the trilaminar endometrial pattern was associated with optimal visualization (79.5% [31/39] *p* = 0.012). In total, 83.3% (5/6) of the cases of diagnosed or suspected endometriosis had unsatisfactory visualization of the JZ (*p* < 0.001) (Table 6). Performing a univariate logistic regression model, considering the unsatisfactory assessment of the JZ as an outcome, we found that the serum estradiol levels (*p* = 0.016) and diagnosed or suspected endometriosis (*p* = 0.004) were the only factors associated with JZ visualization. The joint effect of the variables on the visualization of the JZ was evaluated by multivariate analysis. The results suggest that an increase of 1 unit in the level of serum estradiol represents a 9.9% decrease in the odds of unsatisfactory visualization (odds ratio [OR] = 0.9; 95% confidence interval [95%CI] = 0.814–0.996; *p* = 0.042). On the other hand, the presence of diagnosed or suspected endometriosis increases the odds of unsatisfactory visualization of the JZ by 24 times (OR = 23.7; 95%CI = 1.262–437.057; *p* = 0.034).

**Table 6:** Factors affecting the visualization of the junctional zone (*n* = 60^#^)

Factors	*Visualization of the junctional zone*			*p*-value
	Optimal (*n* = 39)	Satisfatory (*n* = 15)	Unsatisfatory (*n* = 6)	
Estradiol (pg/mL)	59.0(46.0–75.0)	50.0(42.0–84.0)	24.0(20.0–39.0)	0.016* ^(a)^
Endometrial ultrasound characteristics				0.012* ^(b)^
Trilaminar	31(79.5)	6(40.0)	3(50.0)	
Heterogeneous	8 (20.5)	9 (60.0)	3 (50.0)	
Diagnosed or suspected endometriosis	2 (5.1)	6 (40.0)	5 (83.3)	< 0.001* ^(b)^

Notes: ^#^Excluding cases of interobserver and intraobserver disagreement in the visualization of the junctional zone (*n* = 5); results presented as Mdn (P_25_–P_75_) = Mdn, median; P, percentile; and n(%); *p < 0,05; ^(a)^Kruskall-Wallis test, Dunn test with differences between unsatisfactory and optimal visualization (p = 0,013); ^(b)^Chi-squared test.

### Factors Affecting JZ Continuity

We tried to identify the variables that influenced the continuity of the JZ. The interobserver and intraobserver disagreements were solved, and the cases of unsatisfactory visualization were excluded. After the exclusion, 60% (33/55) of the women had a discontinuous JZ. The presence of myomas and of cases of diagnosed or suspected endometriosis are associated with discontinuous JZ (*p* = 0.034 and 0.016 respectively). It was not possible to determine the OR due to the absence of observed frequencies of these variables in continuous JZs (Table 7).

**Table 7:** Factors affecting the continuity of the junctional zone (n = 55^#^)

	JZ continuity		*p*-value
	Discontinuous n(%)	Continuous n(%)	
Myoma n(%)	7(21.2)	0(0)	0.034* ^(a)^
Diagnosed or suspected endometriosis n(%)	8(24.2)	0(0)	0.016* ^(a)^

Notes: ^#^Excluding cases of unsatisfactory visualization of the junctional zone (*n* = 6) and interobserver and intraobserver disagreement regarding continuity of the junctional zone (*n* = 4); results presented as n(%); *p < 0,05; ^(a)^Chi-squared test.

## Discussion

In the present study, the interobserver and intraobserver reproducibility of the JZ visualization was substantial, with values of the Cohen k of 0.635 and 0.884 respectively. Similar results were found in other studies,^14^ ^20^ which indicates that the assessment of the JZ by 3D ultrasound is reproducible enough to be used in the clinical practice. Naftalin et al^14^ proposed that the distinction between optimal and satisfactory visualization of the JZ is subjective. This assumption is corroborated by the present study, in which the largest discrepancies regarding JZ visualization occurred among the cases classified as optimal and satisfactory. On the other hand, the high agreement in cases of JZ visualization classified as unsatisfactory is a relevant result, because JZs with unsatisfactory visualization should not be taken into account for other qualitative or quantitative assessments. These results suggest that a JZ visualization classification system in two categories (satisfactory versus unsatisfactory) is more reproducible.

The interobserver and intraobserver reproducibility of JZ continuity were substantial with the values of Cohen k of 0.753 and 0.816 respectively. To the best of our knowledge, the present was the first study that assessed the reproducibility of this characteristic of the JZ by 3D transvaginal ultrasound. Our findings are relevant and open doors to the inclusion of this evaluation in the clinical practice and to the investigation of its relevance, as it will be discuss later.

The present study showed that estradiol levels and trilaminar endometrial pattern facilitate JZ visualization. The JZ experiences estradiol-mediated cyclic changes consistent with those occurring in the endometrium, and reaches maximal thickness around the 8th and 16th days of the menstrual cycle.^3^ ^21^ ^22^ Thus, higher estradiol levels appear to be associated with a thicker JZ, which facilitates JZ visualization. This finding needs to be proven in future studies. Additionally, estradiol influences the echogenicity of the endometrium, and its role as a confounder cannot be excluded.^23^ In fact, in the present study the trilaminar endometrial pattern was associated with better visualization of the JZ, perhaps because it increases the contrast between the endometrium and the myometrium, which facilitates the achievement of the ideal coronal plane for JZ visualization.

Contrary to other studies,^14^ ^24^ endometrial thickness did not affect the visualization of the JZ. The prevalence of thin endometria (< 5 mm) in our study was of only 24.6%, which may have contributed to this result. As in the study by Naftalin el al,^14^ in the present study we were unable to demonstrate that the menstrual cycle phase affects JZ visualization. However, other studies^25^ ^26^ have shown that the JZ changes between the follicular and luteal phases. One explanation for this result may be the low number of women in the luteal phase in the present study.

Previous studies^12^ ^24^ show higher prevalence of abnormalities in the JZ in women with endometriosis. In the present study, most women with suspected or diagnosed endometriosis had unsatisfactory visualization of the JZ. In fact, the presence of this pathology increases the chance of unsatisfactory visualization of the JZ by ∼ 24 times. Distortions of the normal pelvic anatomy, including the uterus, associated with endometriosis make the acquisition of the ideal coronal plane for the visualization of the JZ difficult, which justifies these findings.^27^ ^28^ On the other hand, the disruption of the normal architecture of the JZ associated with endometriosis makes visualization of the JZ difficult.^12^ ^24^

The prevalence of discontinuous JZs was 60%. The implications of this finding in women with infertility are uncertain. According to the literature,^2^ ^9^ ^10^ ^11^ ^27^ the disruption of the normal architecture of the JZ inevitably alters the coordinated peristaltic activity of the myometrium, interferes with sperm transport and implantation, and affects fertility. Disruption of the JZ also seems to be associated with JZ hyperplasia, endometriosis, and adenomyosis-infertility-related diseases.^12^ ^27^ ^28^

In the present study, discontinuous JZs were associated with suspected or diagnosed endometriosis. Peristaltic hyperactivity of the myometrium, conditioned by JZ disruption, seems to play an important role in the pathophysiology of endometriosis, facilitating retrograde menstruation and implantation of endometrial cells in the pelvic cavity.^2^ ^9^ ^10^ On the other hand, JZ disruption may enable the penetration of endometrial glands into the myometrium and the development of adenomyosis.^9^ ^24^

The prevalence of adenomyosis in women of reproductive age is heterogeneous, ranging from 16% to 66%.^14^ ^29^ Different study methodologies, populations, and diagnostic criteria can explain this disparity. In the present study, the prevalence of adenomyosis was null. This prevalence draws attention to the underdiagnoses of adenomyosis in infertile women. Puente et al^11^ showed that only one in five infertile women had a diagnosis of adenomyosis before the ultrasound evaluation in their study, which reinforces the underdiagnosis of adenomyosis in infertile women. These results highlight the need to make physicians aware of adenomyosis in infertile women and the need to establish reproducible diagnostic ultrasound criteria.^30^

The pathophysiological link between adenomyosis and endometriosis seens to be a disruption of JZ.^11^ ^12^ ^24^ ^27^ ^31^ ^32^ Based on this, the theory of “endometrial-subendometrial myometrium unit disruption disease” was proposed by Tocci et al.^31^ According to this theory, a disruption in the architecture of the JZ seems to constitute the triggering event (causing dysfunction in the uterine peristalsis), and endometriosis and adenomyosis represent phenotypes of “endomyometrial dysfunction” and not distinct pathologies.^31^ Efforts should be made to better describe this entity through 3D transvaginal ultrasound and to assess its impact on women's reproductive functions.

In the present study, the presence of uterine myomas was associated with the presence of discontinuous JZ. In fact, all of the myomas described were classified as type 1 to 3 myomas according to the Fédération Internationale de Gynécologie et d'Obstétrique (International Federation of Gynecology and Obstetrics, FIGO, in the French acronym) classification, which justifies the findings and implications on fertility.^33^

The 3D ultrasound to assess the JZ is cost-effective and noninvasive. The obtained results indicate that the assessment of the visualization and continuity of the JZ by 3D ultrasound is reproducible enough to be used in the clinical practice. The presence of disruption in the architecture of the JZ should alert the clinician to the possibility of the presence of “endometrial-subendometrial myometrium unit disruption disease” due to its association with endometriosis and adenomyosis and, consequently, its implications for women's reproductive and obstetric health.^34^

The assessment of visualization and continuity of the JZ can be criticized regarding its subjectivity, which is a limitation of the present study. However, the same methodology has been used in other studies^14^ ^35^ to assess the reproducibility of JZ visualization, and similar results have been obtained. Another limitation consists in the small sample obtained.

To the best of our knowledge, the present is the first study aiming to assess the JZ through 3D ultrasound in infertile women in Portugal. The study raises new lines of research. One point to develop is the assessment of the impact of JZ disruption on fertility. Long-term follow-up of these women is necessary to evaluate the results of assisted reproductive technology and to relate them to our results.

Thus, due to its reproducibility, the clinical information obtained, and the cost-effectiveness, the evaluation of the JZ through 3D ultrasound should be part of the routine study of infertile women.

## Conclusion

Based on the results obtained from the present study, we emphasize the reproducibility of transvaginal 3D ultrasound assessment of the JZ and the clinical benefits of its inclusion in the routine evaluation of infertile women.
